# Supplementary figures and images for: Heterogeneous non-canonical nucleosomes predominate in yeast cells in situ
Source: eLife. 2023 Jul 28;12:RP87672. doi: 10.7554/eLife.87672 (PMC10382156; doi:10.7554/eLife.87672)

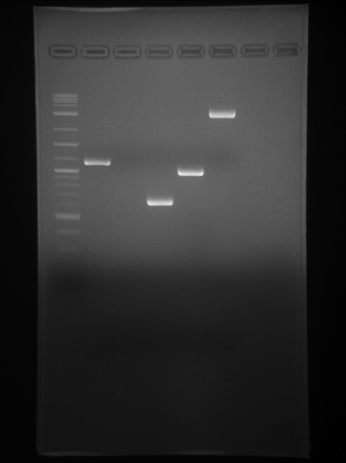

Supplement: Figure 3—figure supplement 2—source data 1. [file elife-87672-fig3-figsupp2-data1.zip › Figure 3-Figure Supplement 2-Source Data 1/Figure 3-Figure Supplement 2-Source Data 1-Raw.tif]

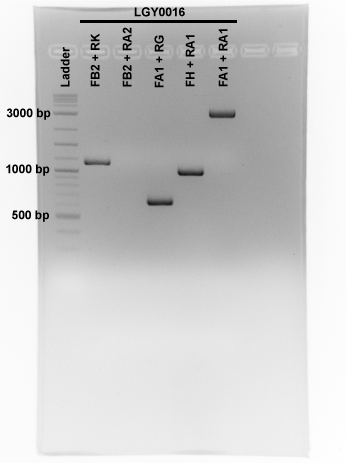

Supplement: Figure 3—figure supplement 2—source data 1. [file elife-87672-fig3-figsupp2-data1.zip › Figure 3-Figure Supplement 2-Source Data 1/Figure 3-Figure Supplement 2-Source Data 1-Labelled.png]

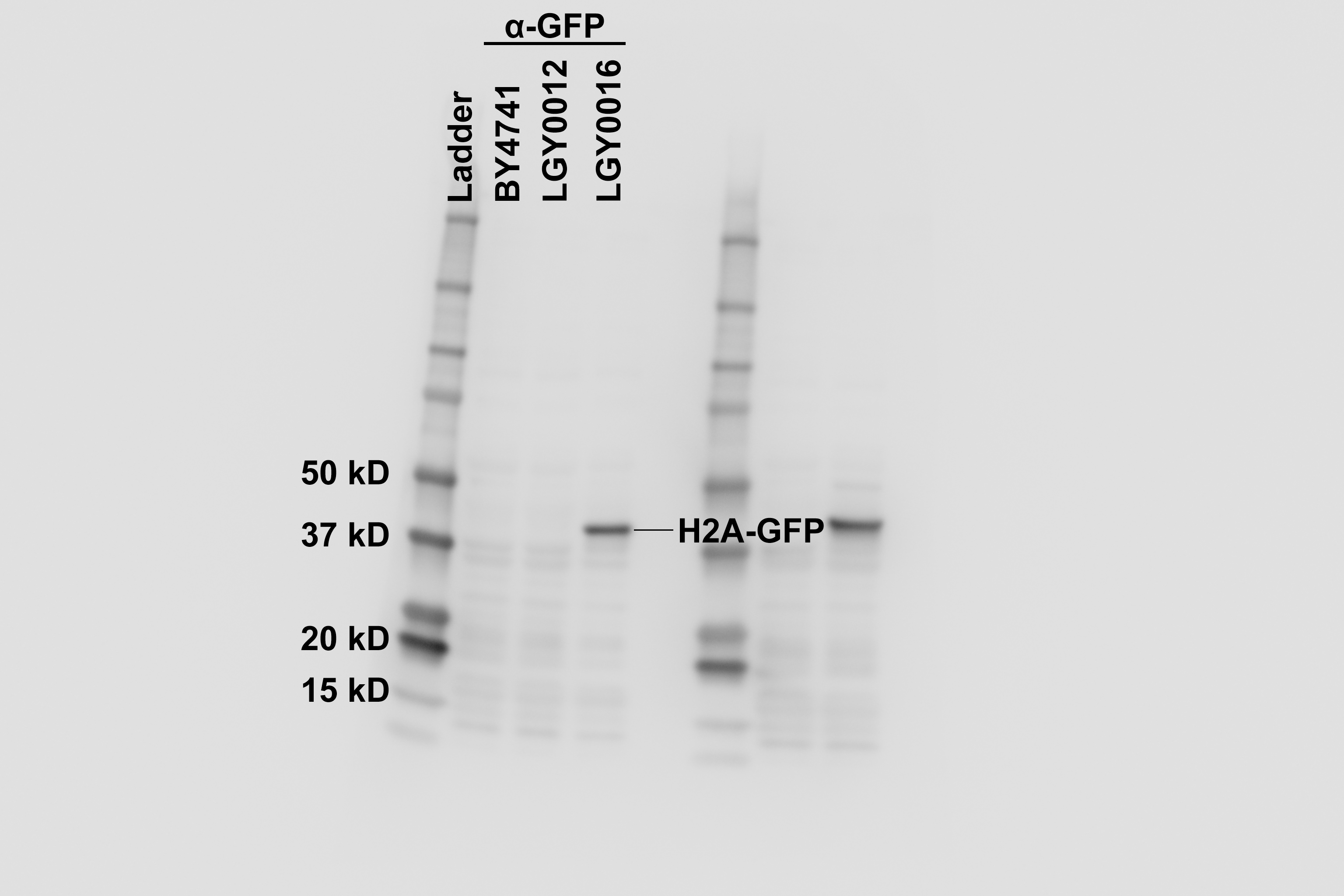

Supplement: Figure 3—figure supplement 2—source data 2. — The unlabeled bands on the right were from a previous attempt for immunoblot analysis of strain LGY0015. [file elife-87672-fig3-figsupp2-data2.zip › Figure 3-Figure Supplement 2-Source Data 2/Figure 3-Figure Supplement 2-Source Data 2-Labelled.png]

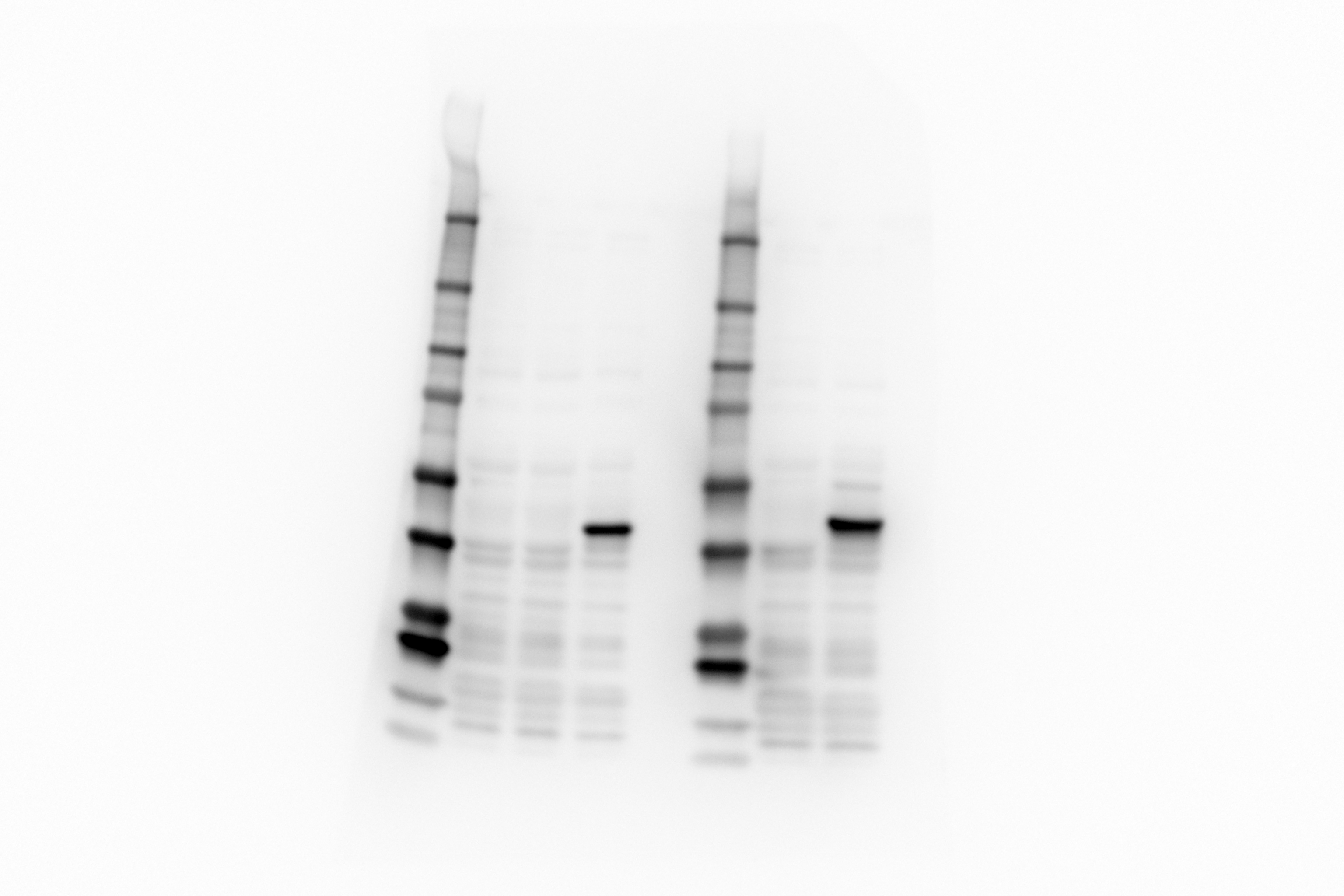

Supplement: Figure 3—figure supplement 2—source data 2. — The unlabeled bands on the right were from a previous attempt for immunoblot analysis of strain LGY0015. [file elife-87672-fig3-figsupp2-data2.zip › Figure 3-Figure Supplement 2-Source Data 2/Figure 3-Figure Supplement 2-Source Data 2-Raw.tif]

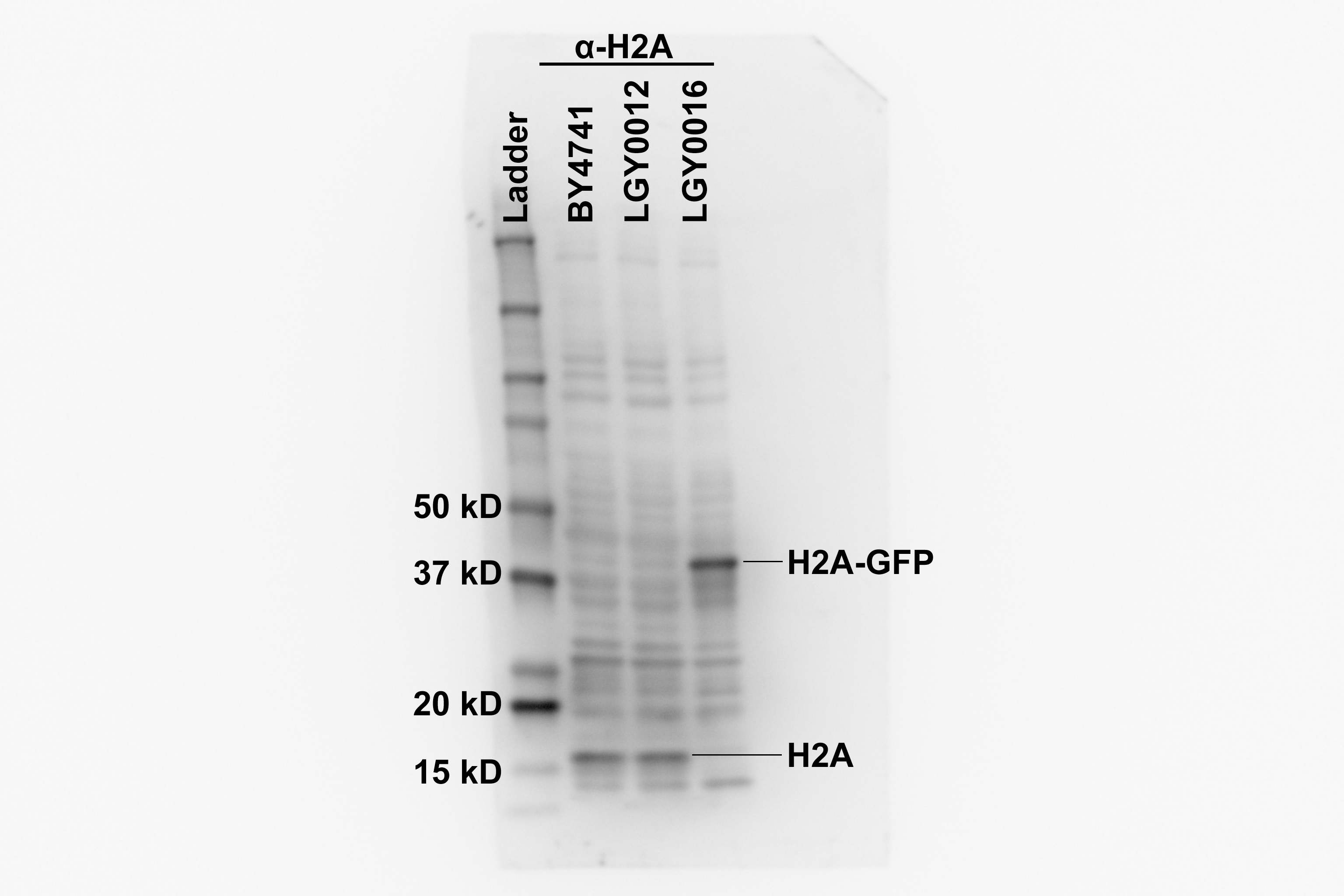

Supplement: Figure 3—figure supplement 2—source data 3. [file elife-87672-fig3-figsupp2-data3.zip › Figure 3-Figure Supplement 2-Source Data 3/Figure 3-Figure Supplement 2-Source Data 3-Labelled.png]

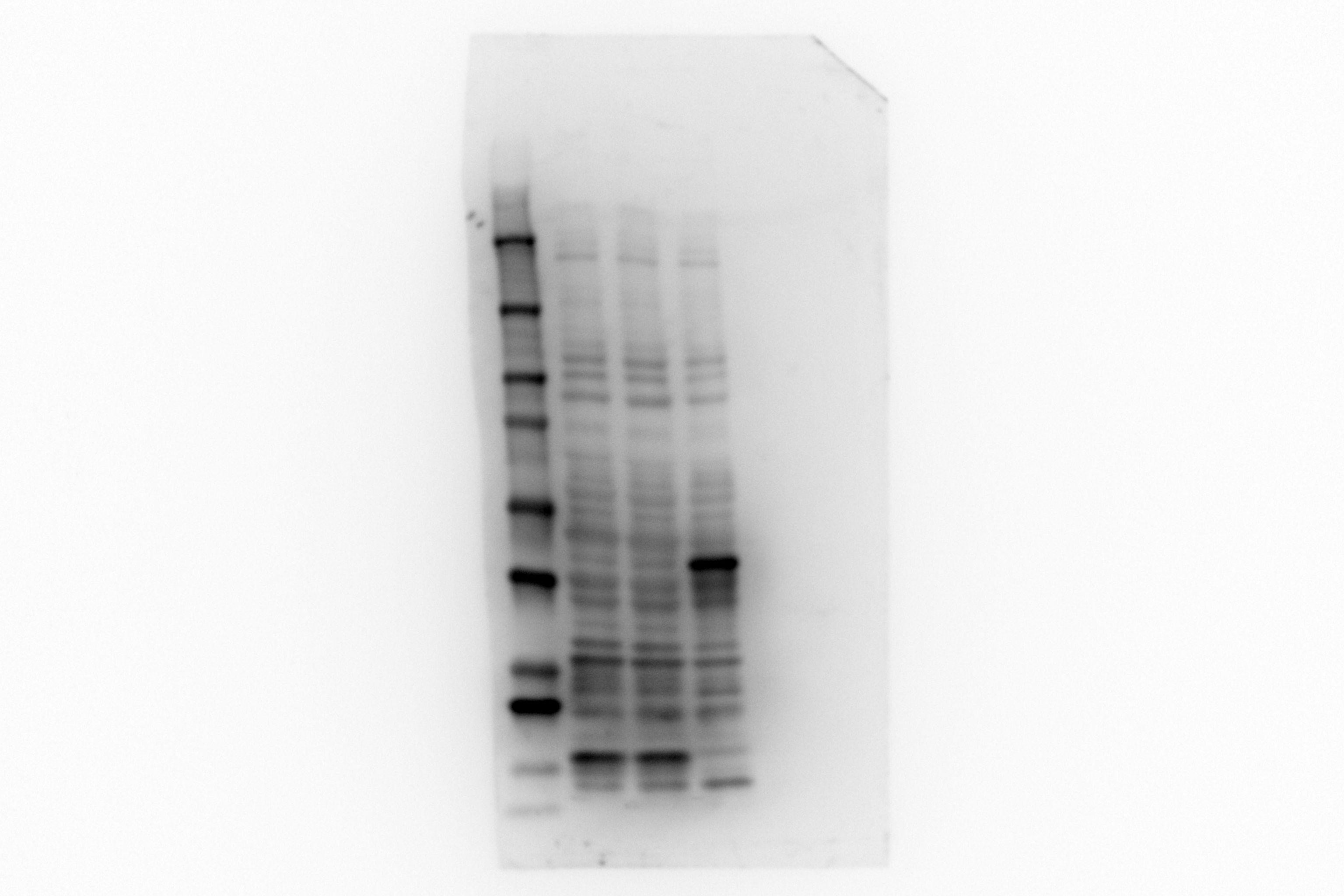

Supplement: Figure 3—figure supplement 2—source data 3. [file elife-87672-fig3-figsupp2-data3.zip › Figure 3-Figure Supplement 2-Source Data 3/Figure 3-Figure Supplement 2-Source Data 3-Raw.tif]

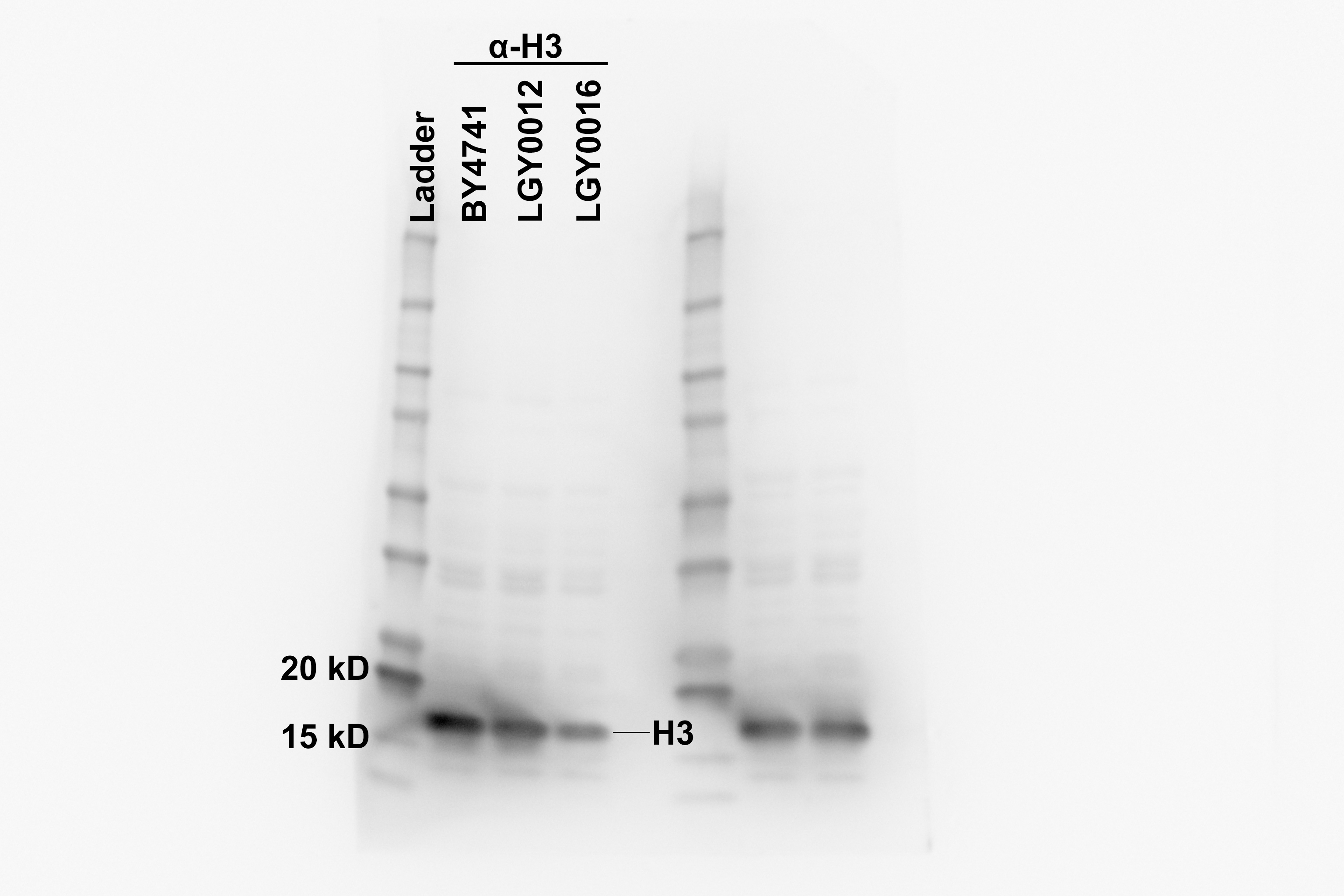

Supplement: Figure 3—figure supplement 2—source data 4. — The unlabeled bands on the right were from a previous attempt for immunoblot analysis of strain LGY0015. [file elife-87672-fig3-figsupp2-data4.zip › Figure 3-Figure Supplement 2-Source Data 4/Figure 3-Figure Supplement 2-Source Data 4-Labelled.png]

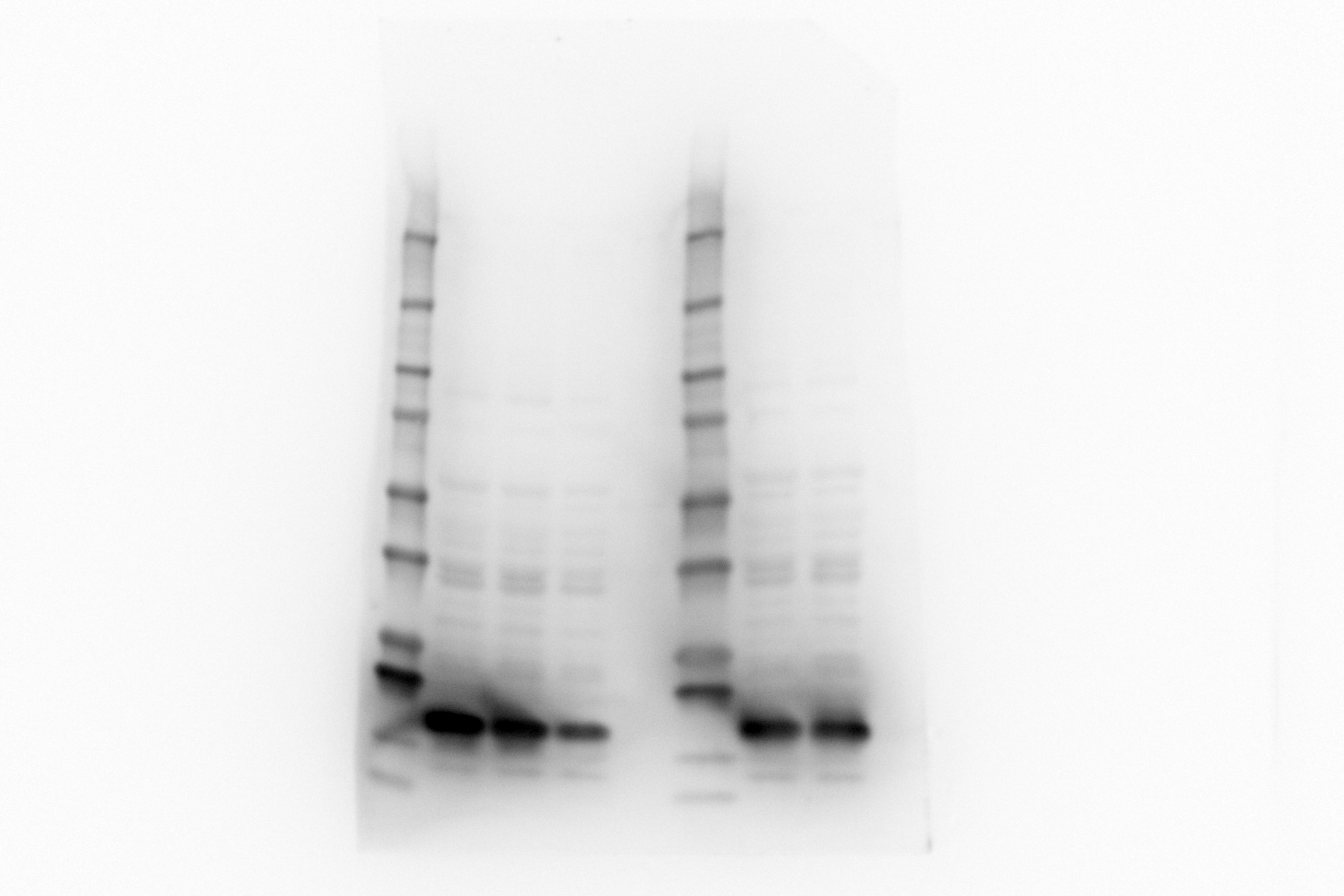

Supplement: Figure 3—figure supplement 2—source data 4. — The unlabeled bands on the right were from a previous attempt for immunoblot analysis of strain LGY0015. [file elife-87672-fig3-figsupp2-data4.zip › Figure 3-Figure Supplement 2-Source Data 4/Figure 3-Figure Supplement 2-Source Data 4-Raw.tif]

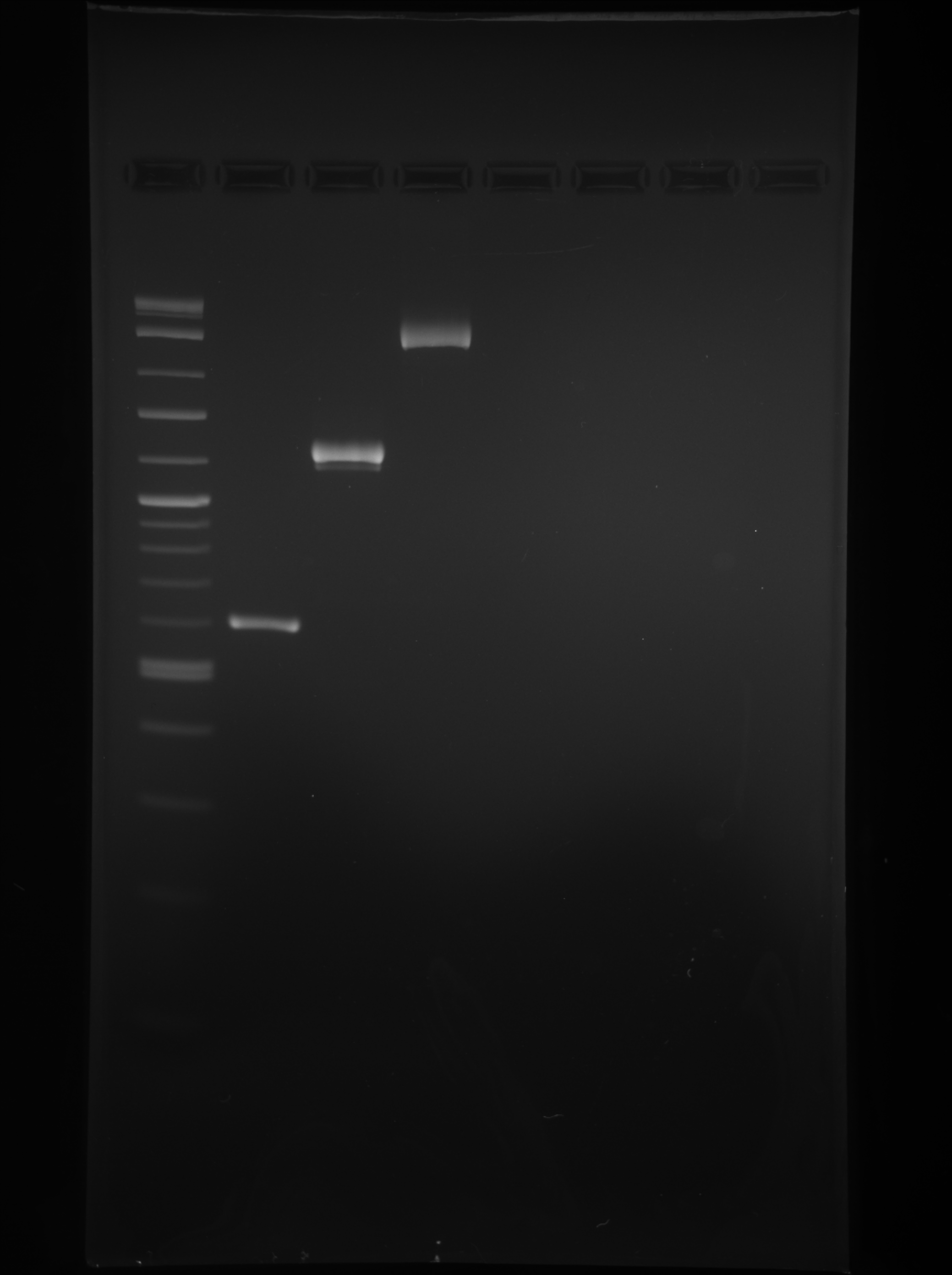

Supplement: Figure 3—figure supplement 2—source data 6. [file elife-87672-fig3-figsupp2-data6.zip › Figure 3-Figure Supplement 2-Source Data 6/Figure 3-Figure Supplement 2-Source Data 6-Raw.tif]

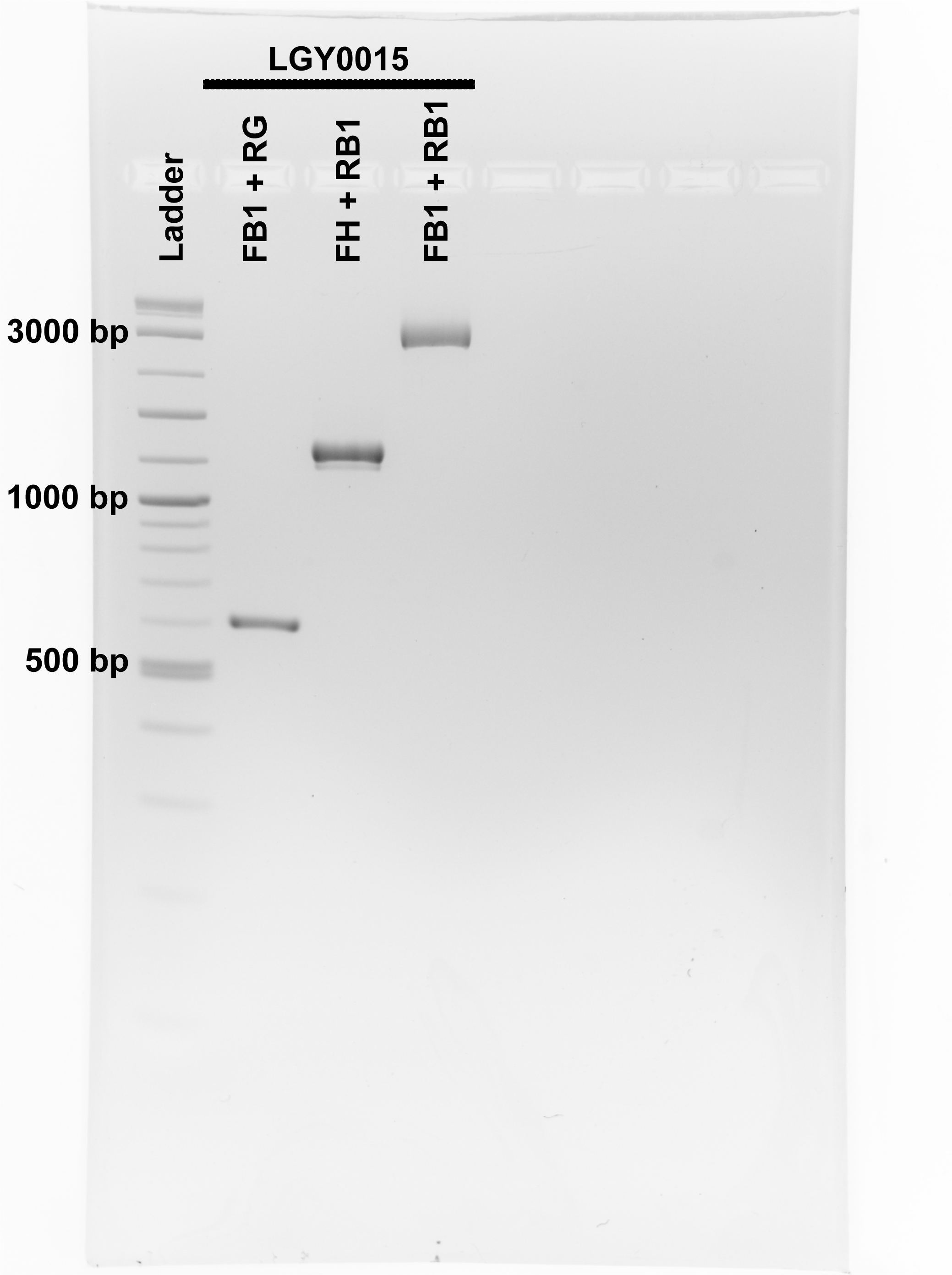

Supplement: Figure 3—figure supplement 2—source data 6. [file elife-87672-fig3-figsupp2-data6.zip › Figure 3-Figure Supplement 2-Source Data 6/Figure 3-Figure Supplement 2-Source Data 6-Labelled.png]

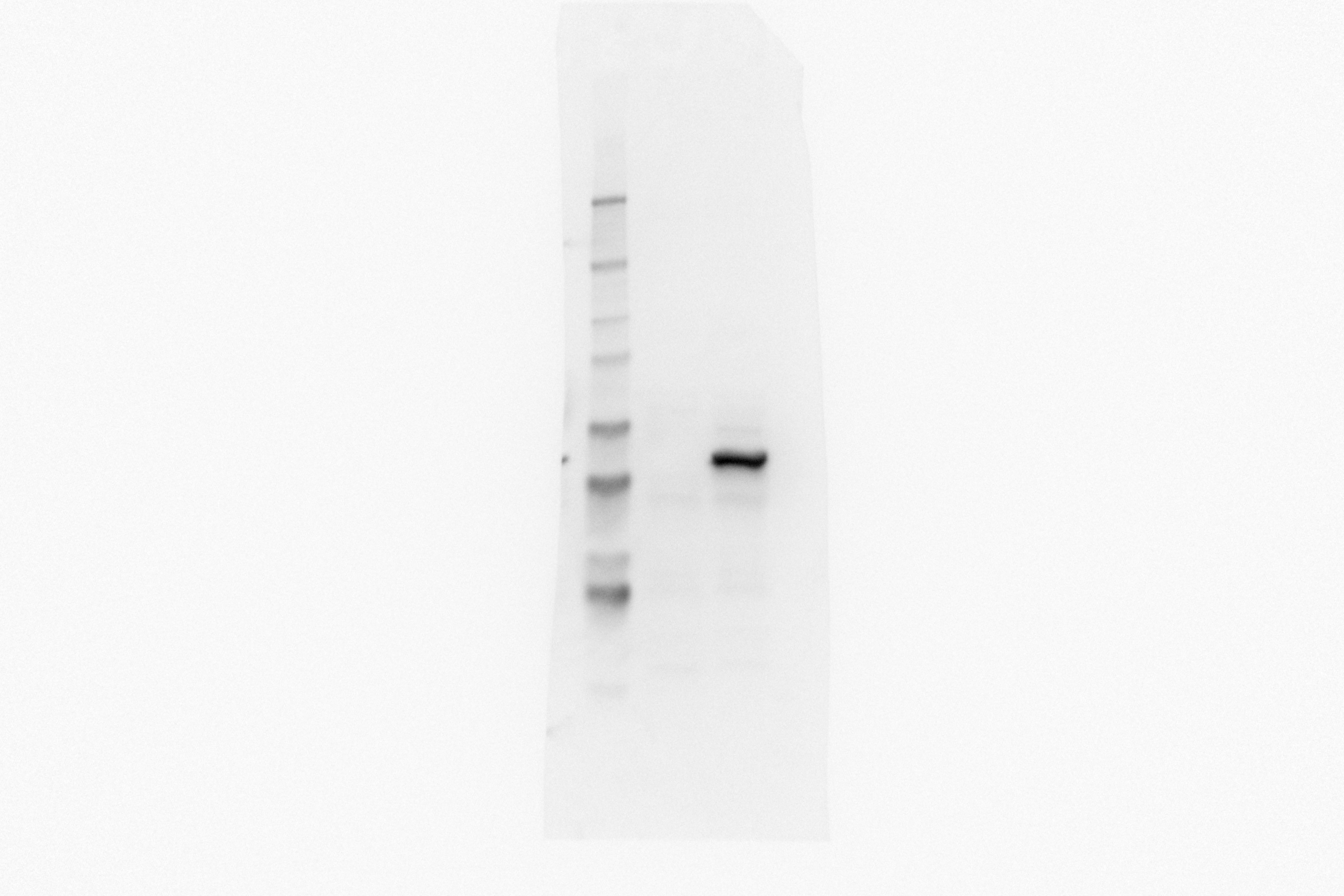

Supplement: Figure 3—figure supplement 2—source data 7. [file elife-87672-fig3-figsupp2-data7.zip › Figure 3-Figure Supplement 2-Source Data 7/Figure 3-Figure Supplement 2-Source Data 7-Raw.tif]

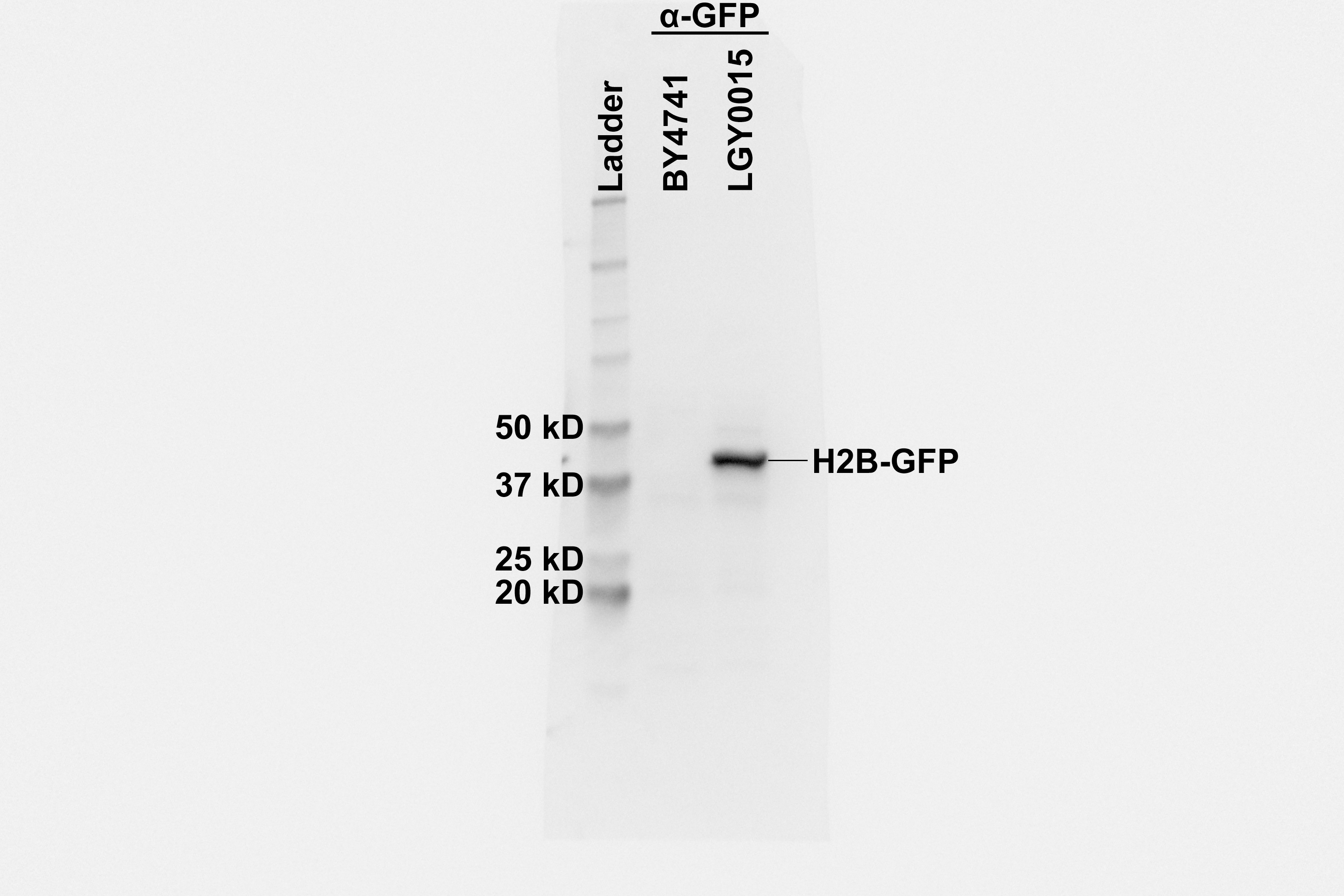

Supplement: Figure 3—figure supplement 2—source data 7. [file elife-87672-fig3-figsupp2-data7.zip › Figure 3-Figure Supplement 2-Source Data 7/Figure 3-Figure Supplement 2-Source Data 7-Labelled.png]

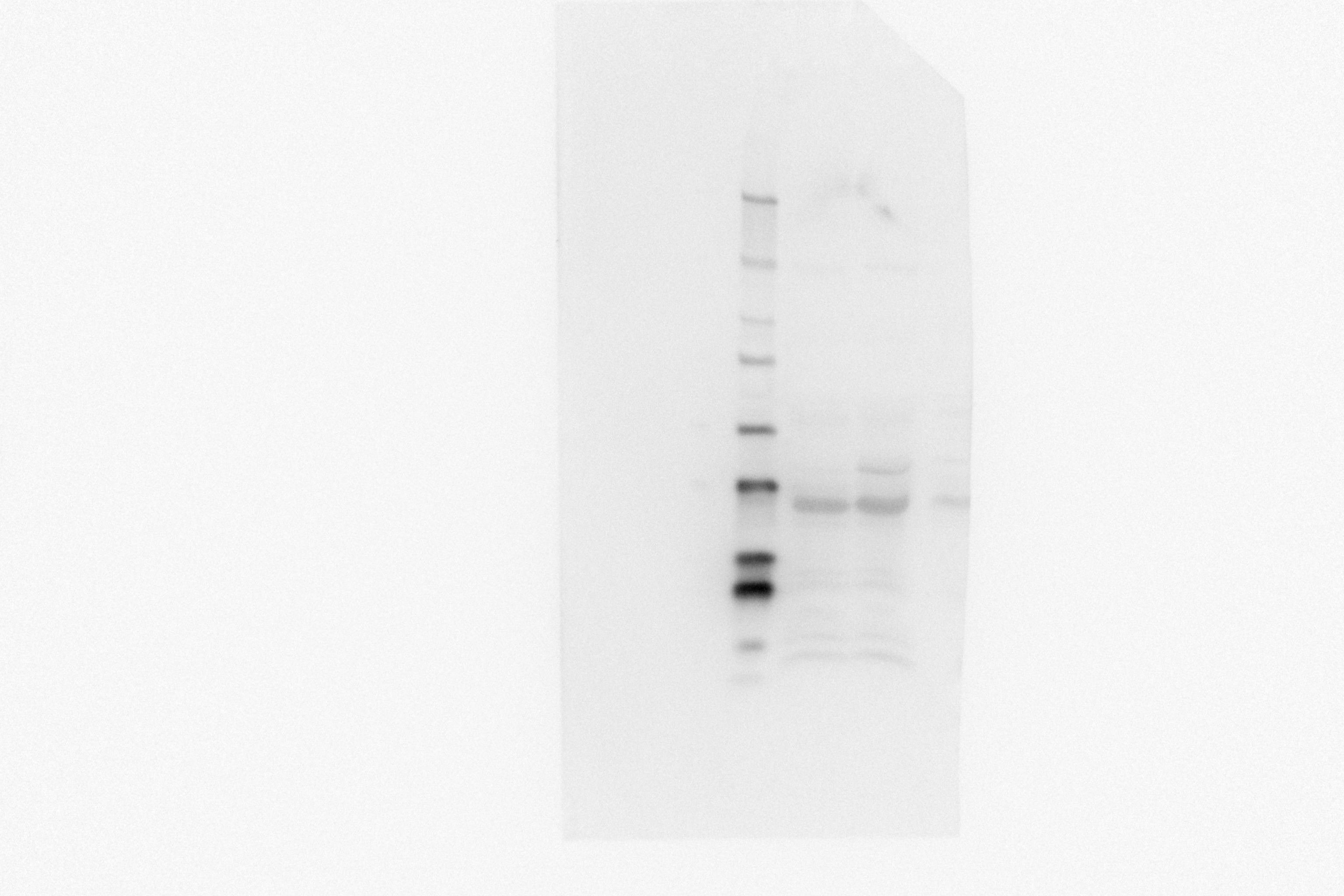

Supplement: Figure 3—figure supplement 2—source data 8. [file elife-87672-fig3-figsupp2-data8.zip › Figure 3-Figure Supplement 2-Source Data 8/Figure 3-Figure Supplement 2-Source Data 8-Raw.tif]

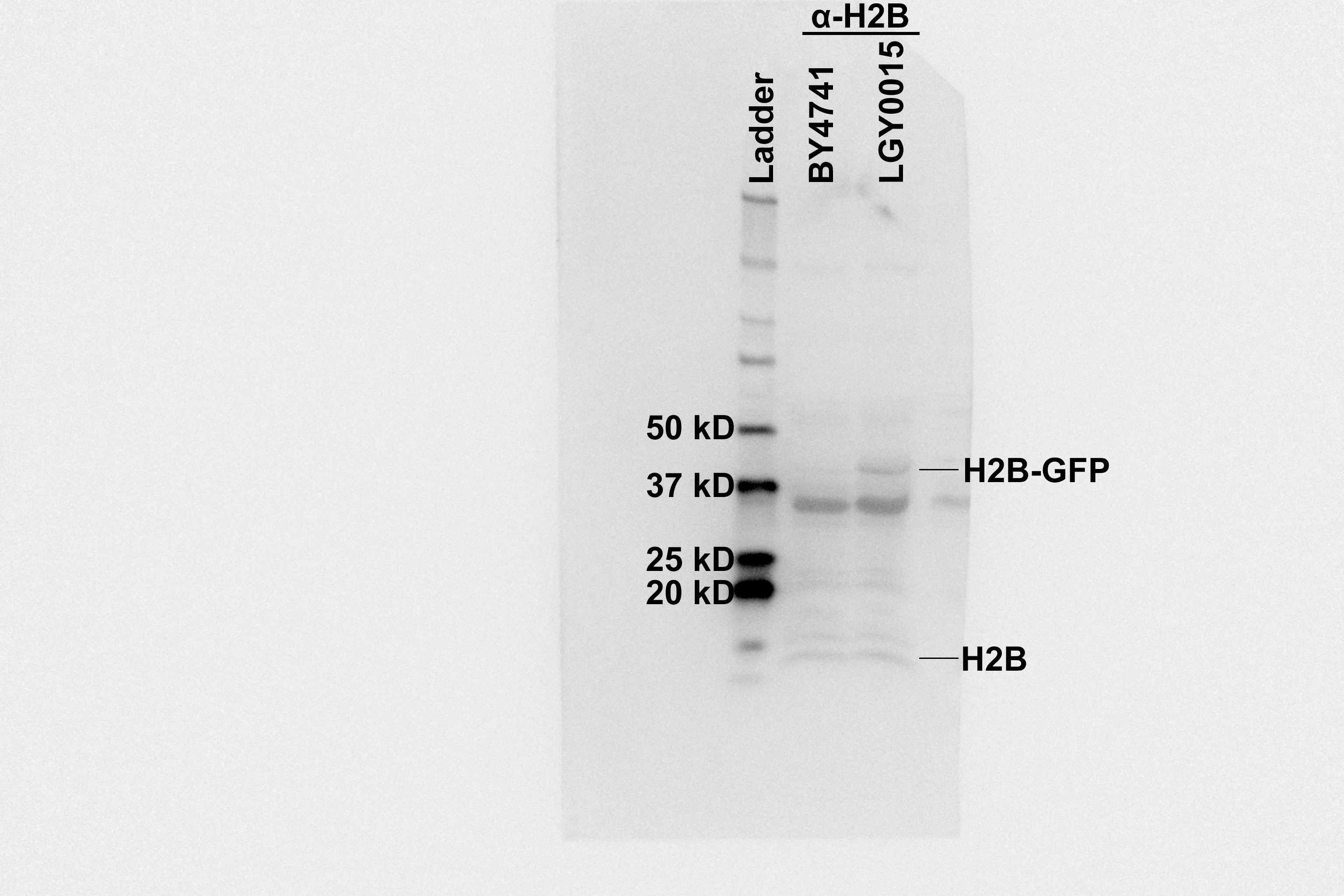

Supplement: Figure 3—figure supplement 2—source data 8. [file elife-87672-fig3-figsupp2-data8.zip › Figure 3-Figure Supplement 2-Source Data 8/Figure 3-Figure Supplement 2-Source Data 8-Labelled.png]

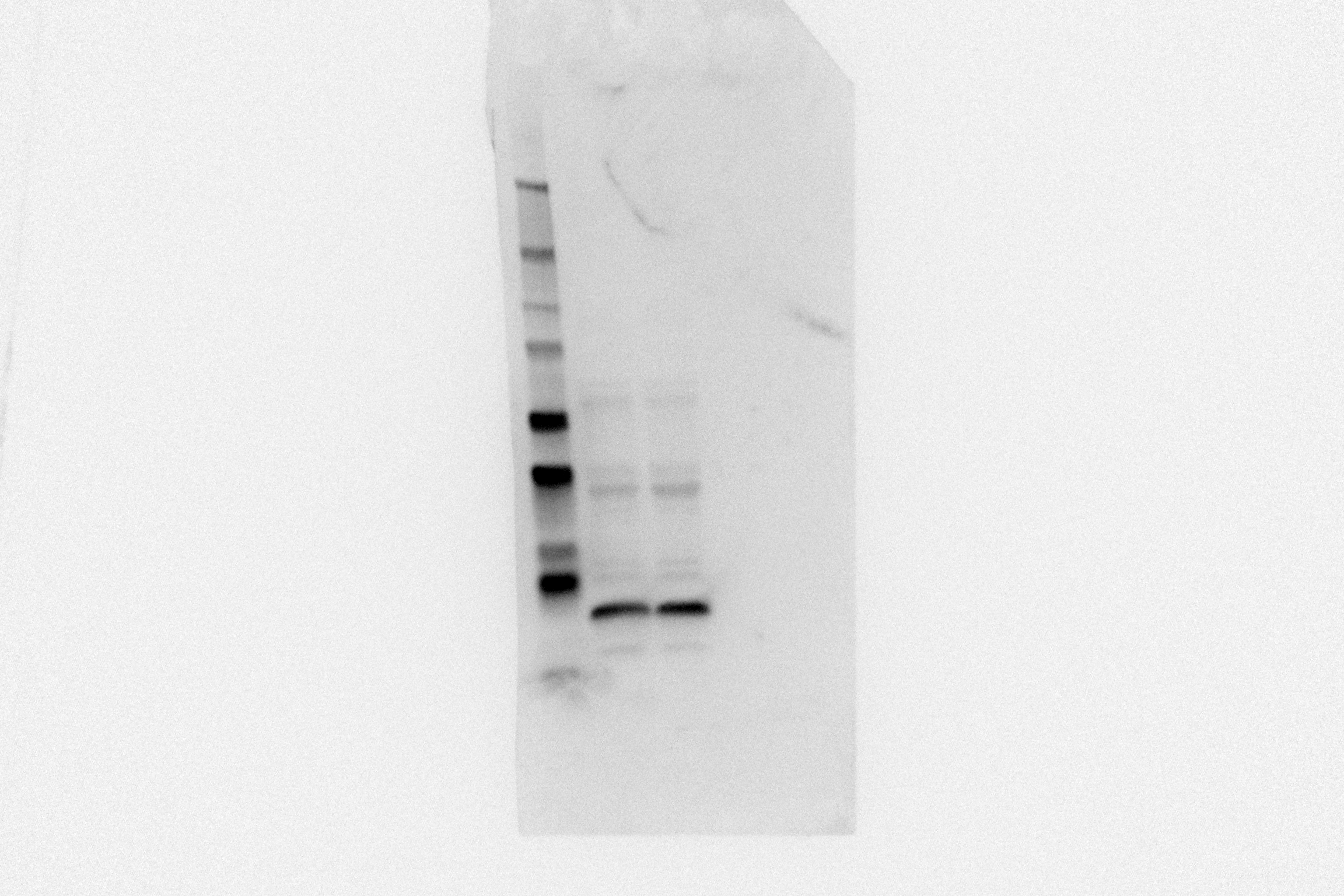

Supplement: Figure 3—figure supplement 2—source data 9. [file elife-87672-fig3-figsupp2-data9.zip › Figure 3-Figure Supplement 2-Source Data 9/Figure 3-Figure Supplement 2-Source Data 9-Raw.tif]

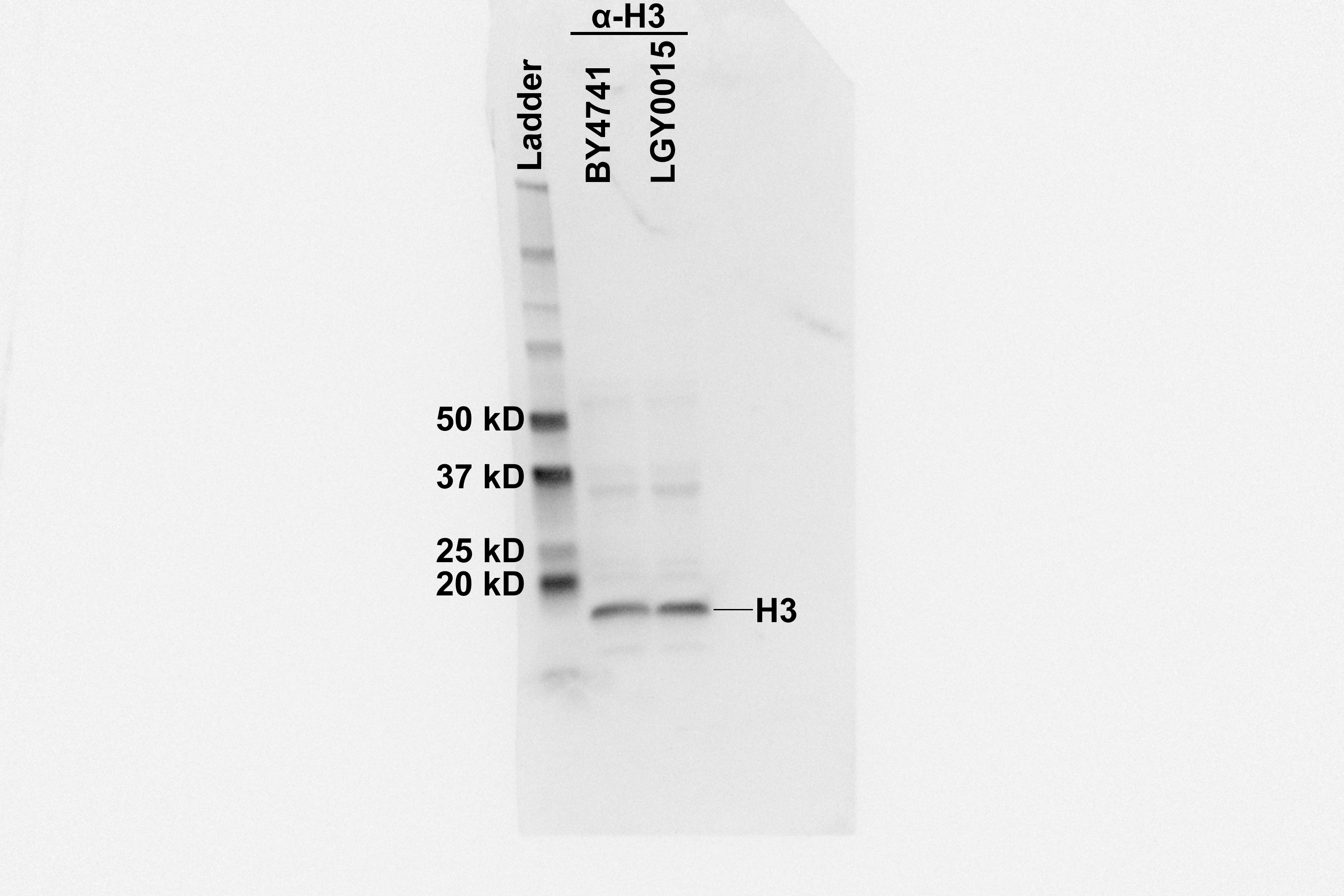

Supplement: Figure 3—figure supplement 2—source data 9. [file elife-87672-fig3-figsupp2-data9.zip › Figure 3-Figure Supplement 2-Source Data 9/Figure 3-Figure Supplement 2-Source Data 9-Labelled.png]

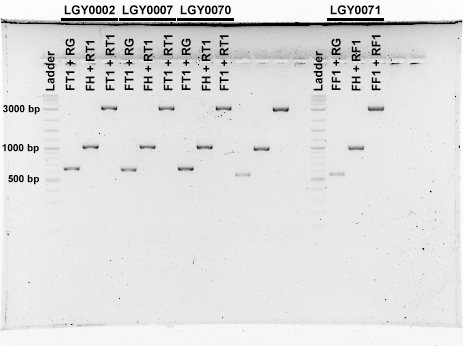

Supplement: Figure 4—figure supplement 7—source data 1. — This data was also used in Figure 4—figure supplement 8, panel B. The unlabeled bands between the HHT1 and the HHF1 bands were duplicate loadings of LGY0071 genomic DNA PCR amplicons. [file elife-87672-fig4-figsupp7-data1.zip › Figure 4-Figure Supplement 7-Source Data 1/Figure 4-Figure Supplement 7-Source Data 1-Labelled.png]

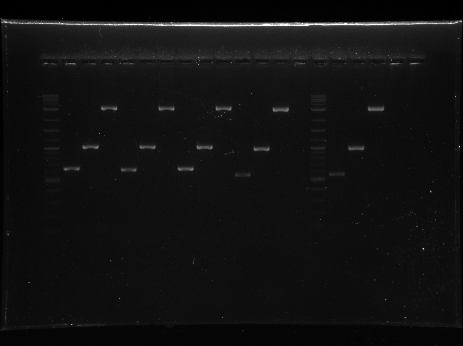

Supplement: Figure 4—figure supplement 7—source data 1. — This data was also used in Figure 4—figure supplement 8, panel B. The unlabeled bands between the HHT1 and the HHF1 bands were duplicate loadings of LGY0071 genomic DNA PCR amplicons. [file elife-87672-fig4-figsupp7-data1.zip › Figure 4-Figure Supplement 7-Source Data 1/Figure 4-Figure Supplement 7-Source Data 1-Raw.tif]

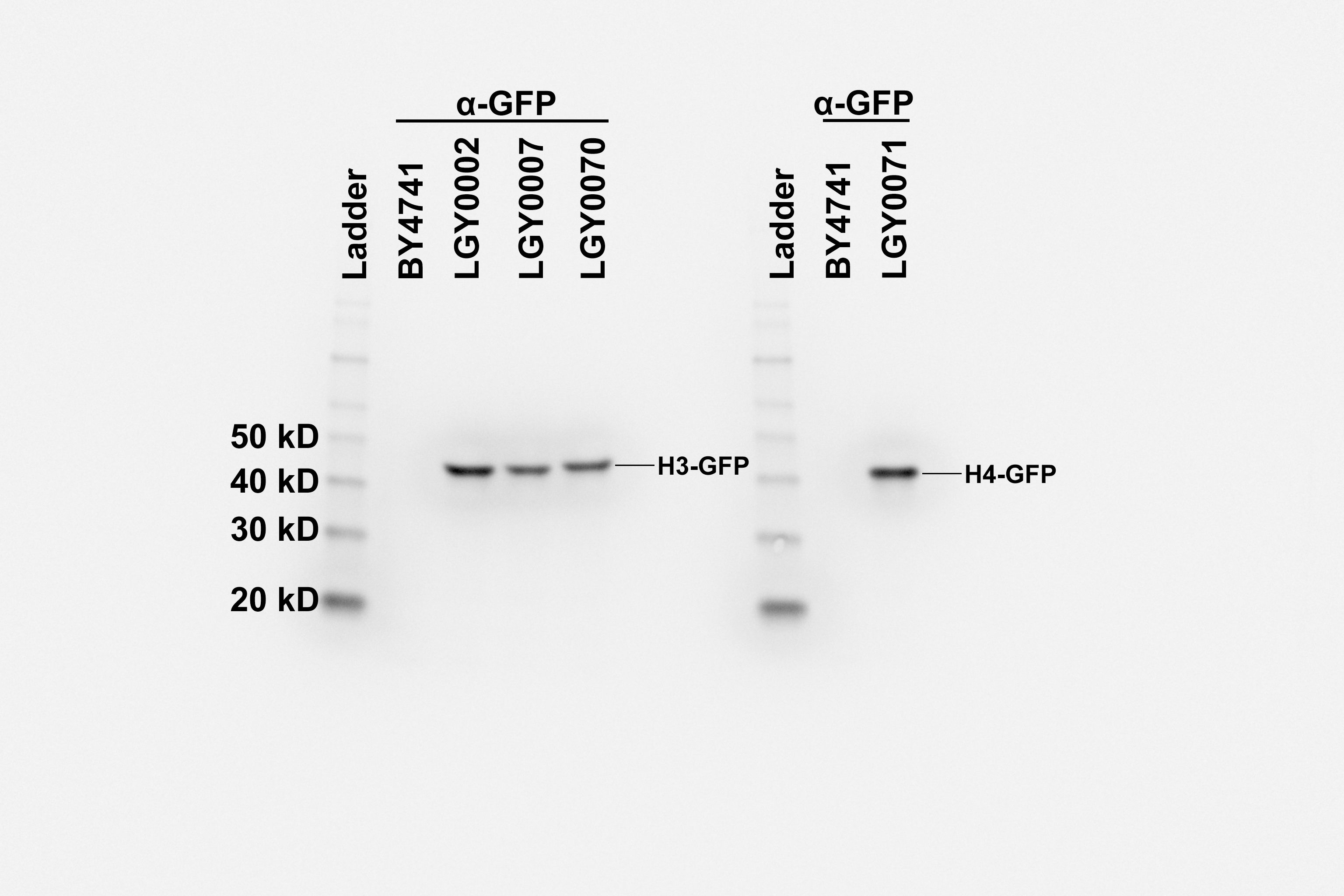

Supplement: Figure 4—figure supplement 7—source data 2. — This data was also used in Figure 4—figure supplement 8, panel C. [file elife-87672-fig4-figsupp7-data2.zip › Figure 4-Figure Supplement 7-Source Data 2/Figure 4-Figure Supplement 7-Source Data 2-Labelled.png]

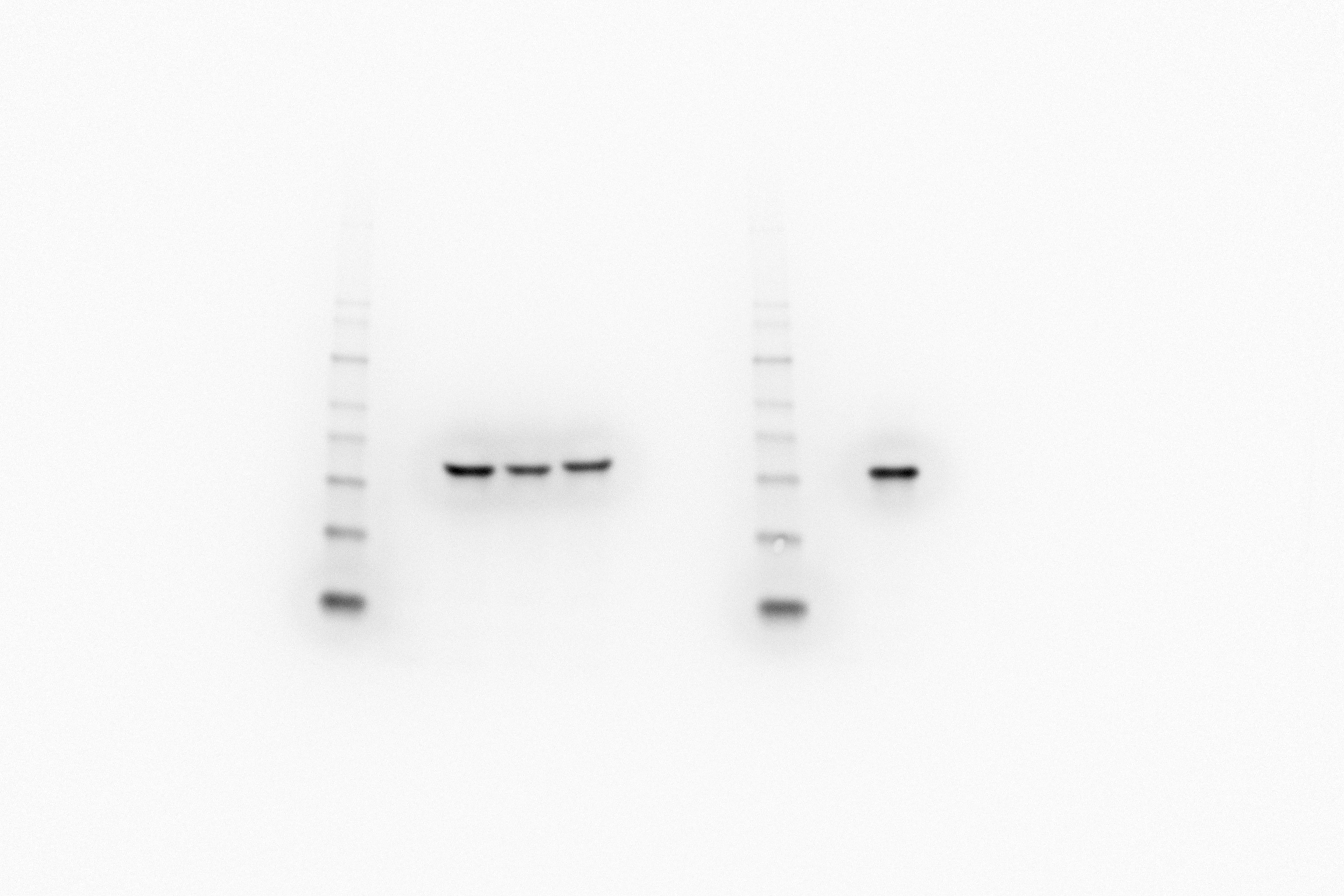

Supplement: Figure 4—figure supplement 7—source data 2. — This data was also used in Figure 4—figure supplement 8, panel C. [file elife-87672-fig4-figsupp7-data2.zip › Figure 4-Figure Supplement 7-Source Data 2/Figure 4-Figure Supplement 7-Source Data 2-Raw.tif]

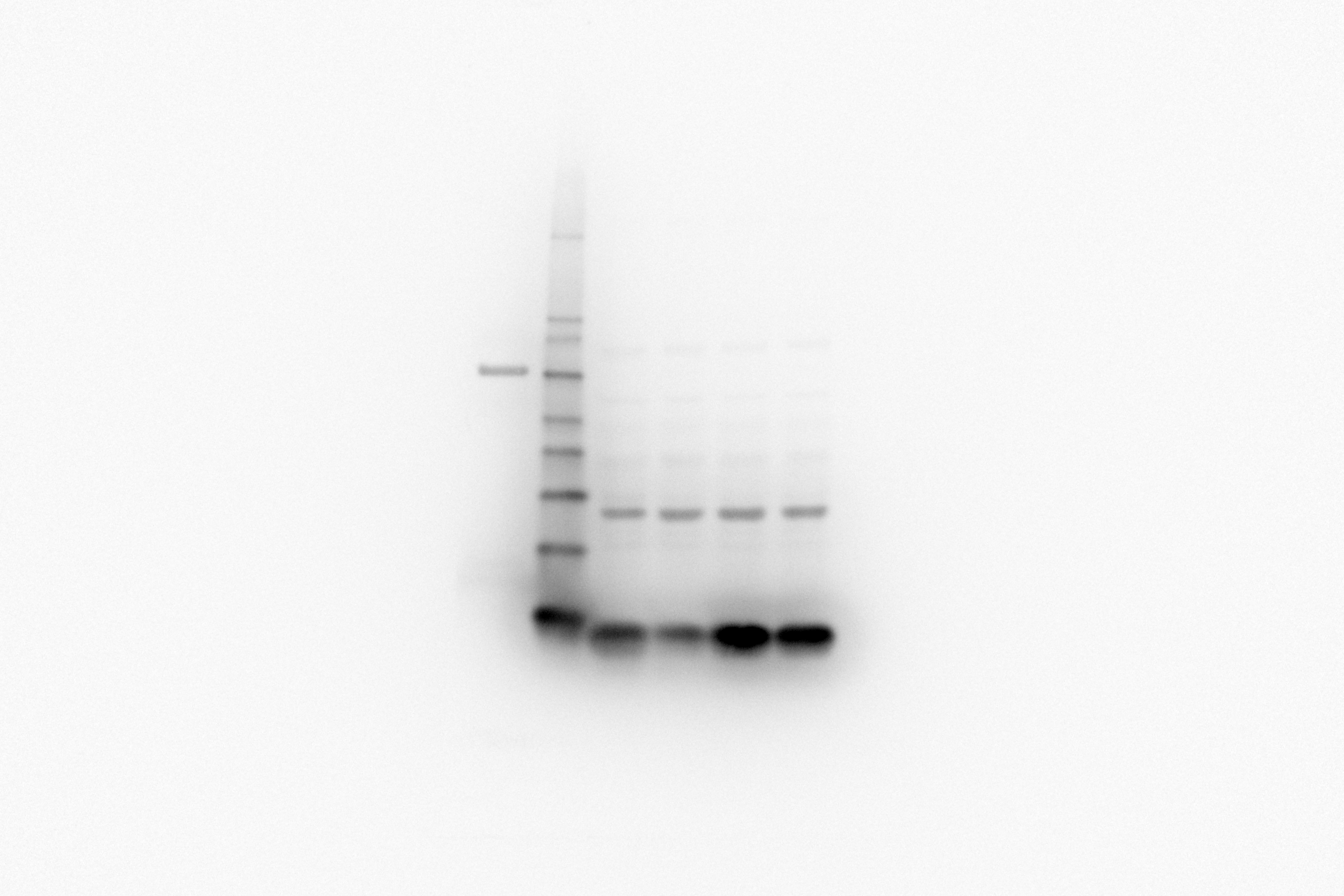

Supplement: Figure 4—figure supplement 7—source data 3. — H3-GFP was expected to appear between 40 kDa and 50 kDa in these strains, but the antibody failed to detect the fusion protein. [file elife-87672-fig4-figsupp7-data3.zip › Figure 4-Figure Supplement 7-Source Data 3/Figure 4-Figure Supplement 7-Source Data 3-Raw.tif]

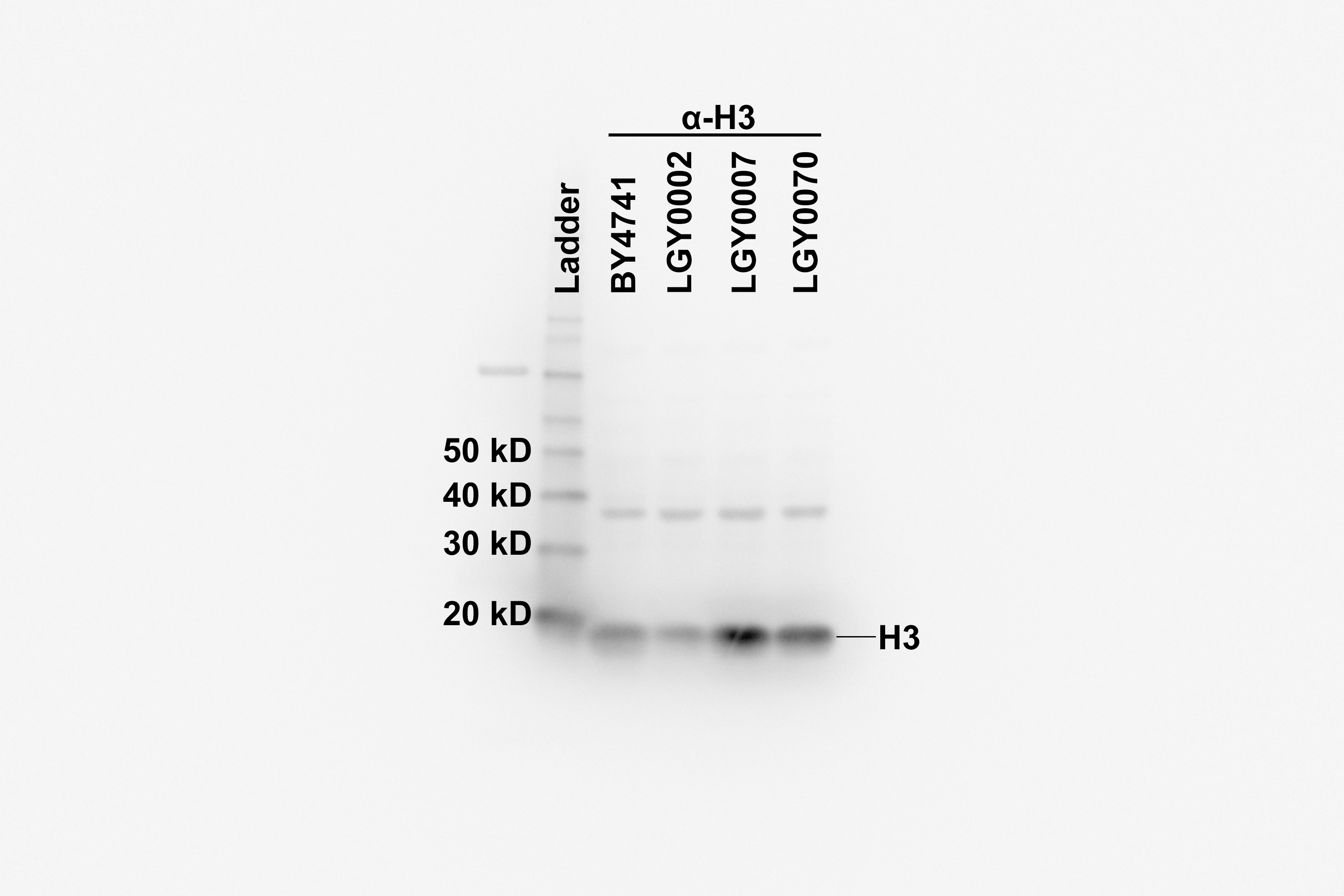

Supplement: Figure 4—figure supplement 7—source data 3. — H3-GFP was expected to appear between 40 kDa and 50 kDa in these strains, but the antibody failed to detect the fusion protein. [file elife-87672-fig4-figsupp7-data3.zip › Figure 4-Figure Supplement 7-Source Data 3/Figure 4-Figure Supplement 7-Source Data 3-Labelled.png]

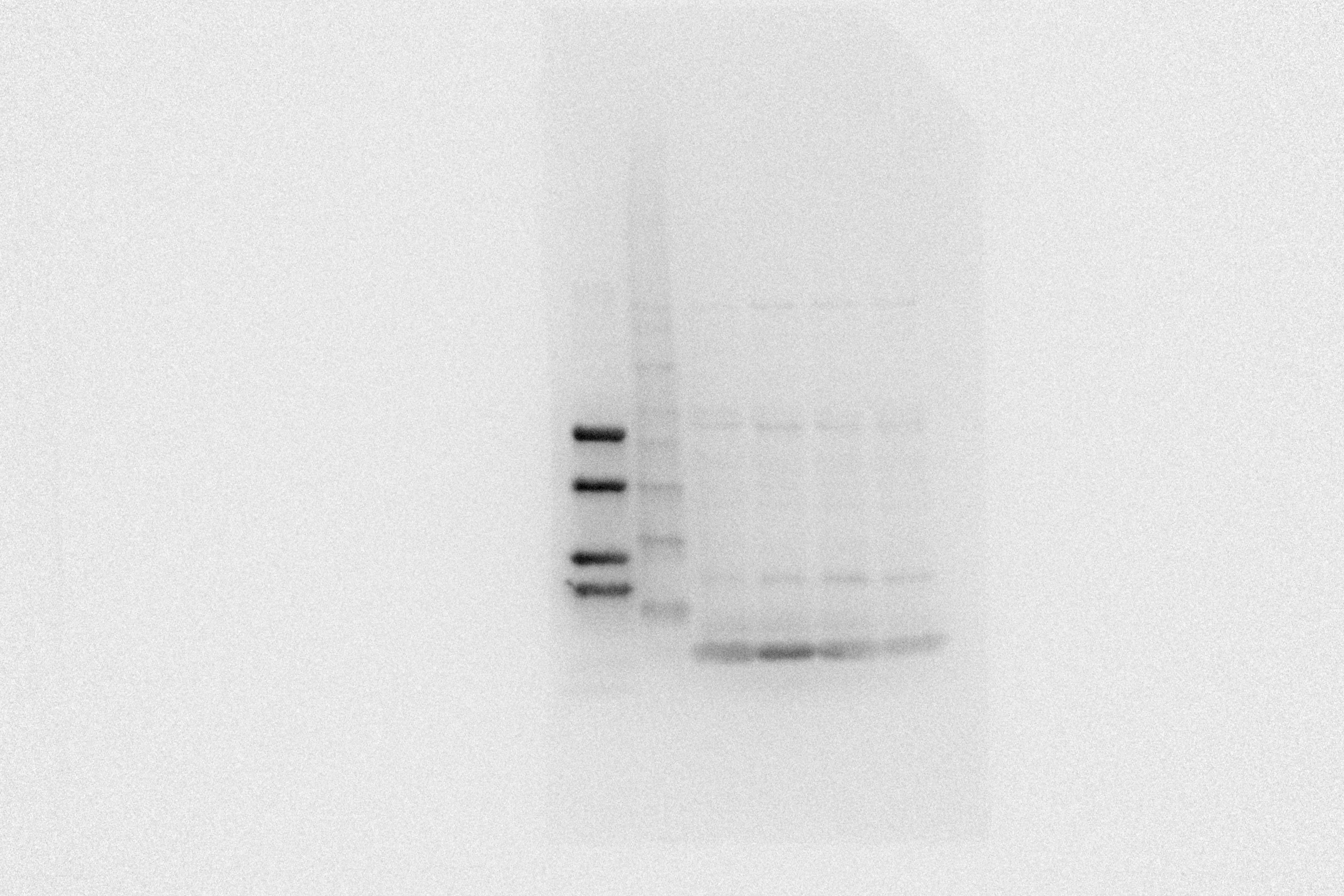

Supplement: Figure 4—figure supplement 7—source data 4. [file elife-87672-fig4-figsupp7-data4.zip › Figure 4-Figure Supplement 7-Source Data 4/Figure 4-Figure Supplement 7-Source Data 4-Raw.tif]

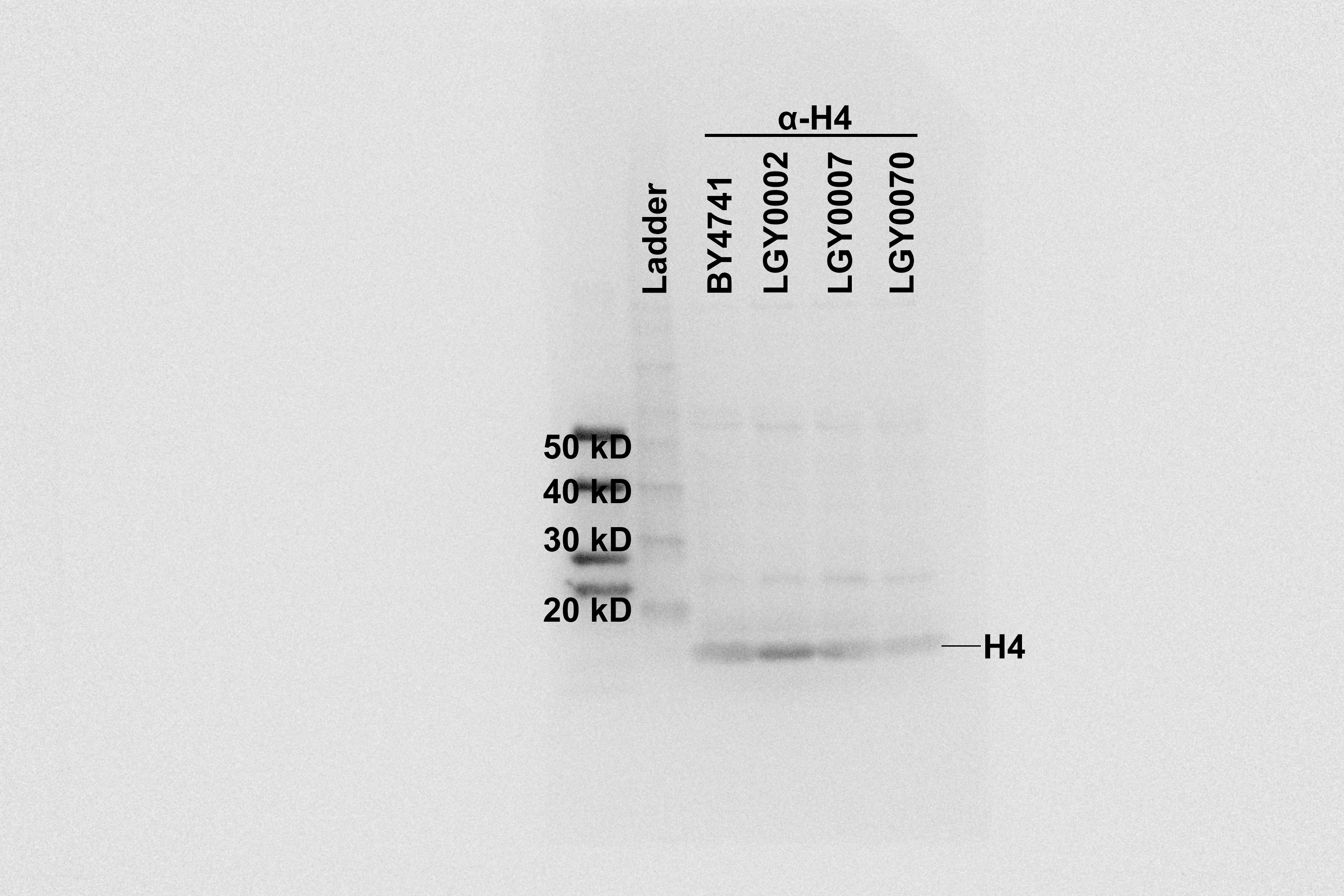

Supplement: Figure 4—figure supplement 7—source data 4. [file elife-87672-fig4-figsupp7-data4.zip › Figure 4-Figure Supplement 7-Source Data 4/Figure 4-Figure Supplement 7-Source Data 4-Labelled.png]

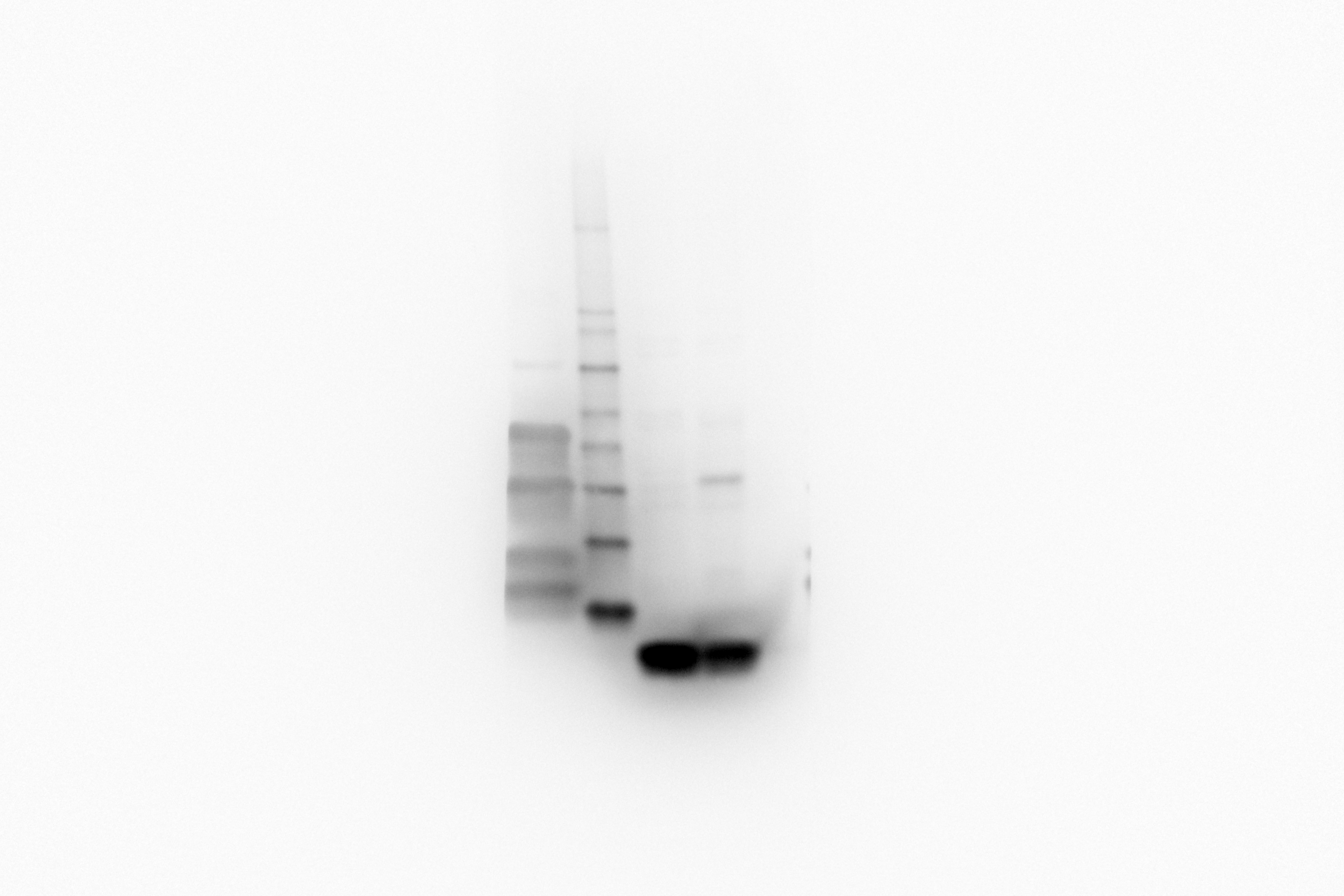

Supplement: Figure 4—figure supplement 8—source data 1. [file elife-87672-fig4-figsupp8-data1.zip › Figure 4-Figure Supplement 8-Source Data 1/Figure 4-Figure Supplement 8-Source Data 1-Raw.tif]

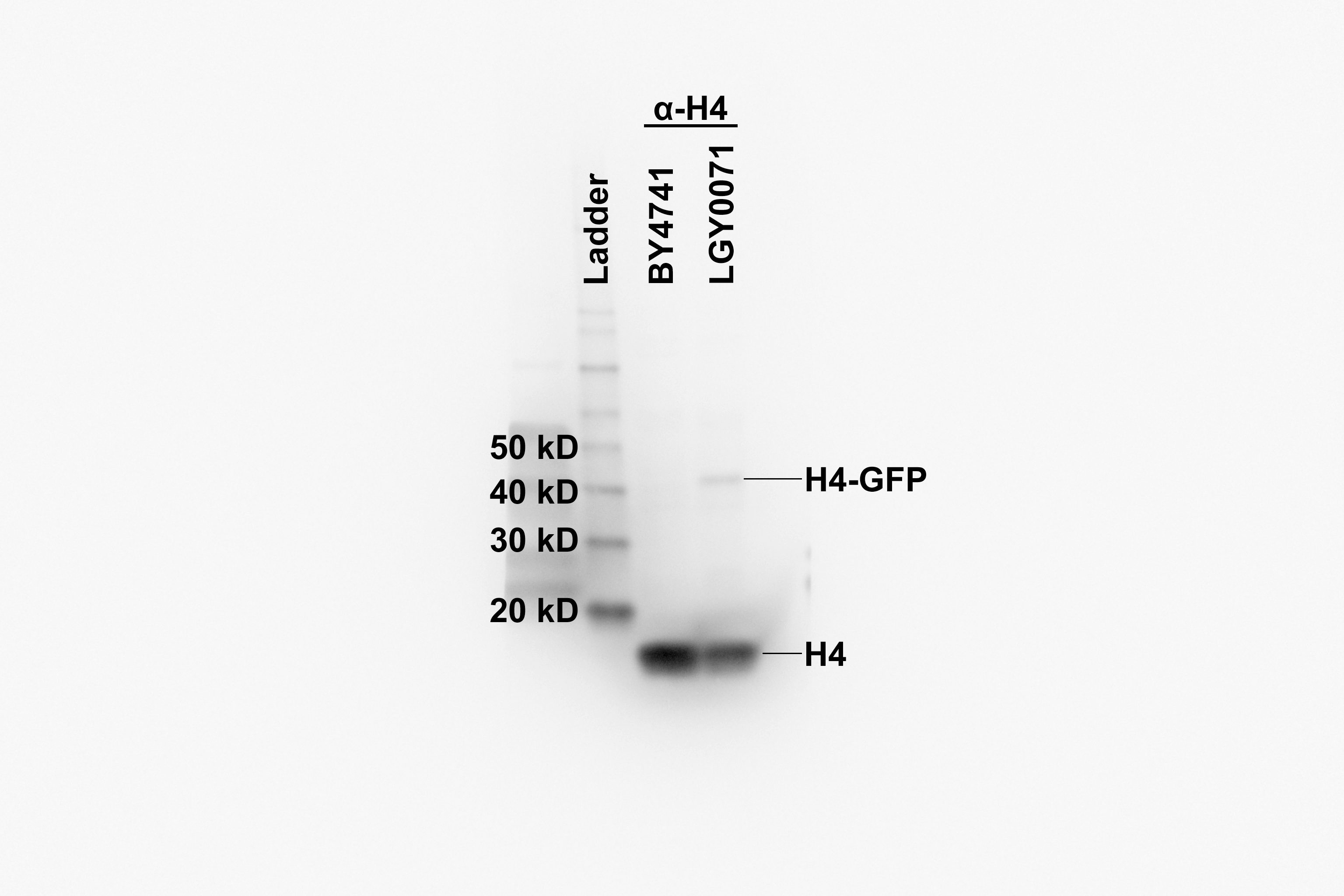

Supplement: Figure 4—figure supplement 8—source data 1. [file elife-87672-fig4-figsupp8-data1.zip › Figure 4-Figure Supplement 8-Source Data 1/Figure 4-Figure Supplement 8-Source Data 1-Labelled.png]

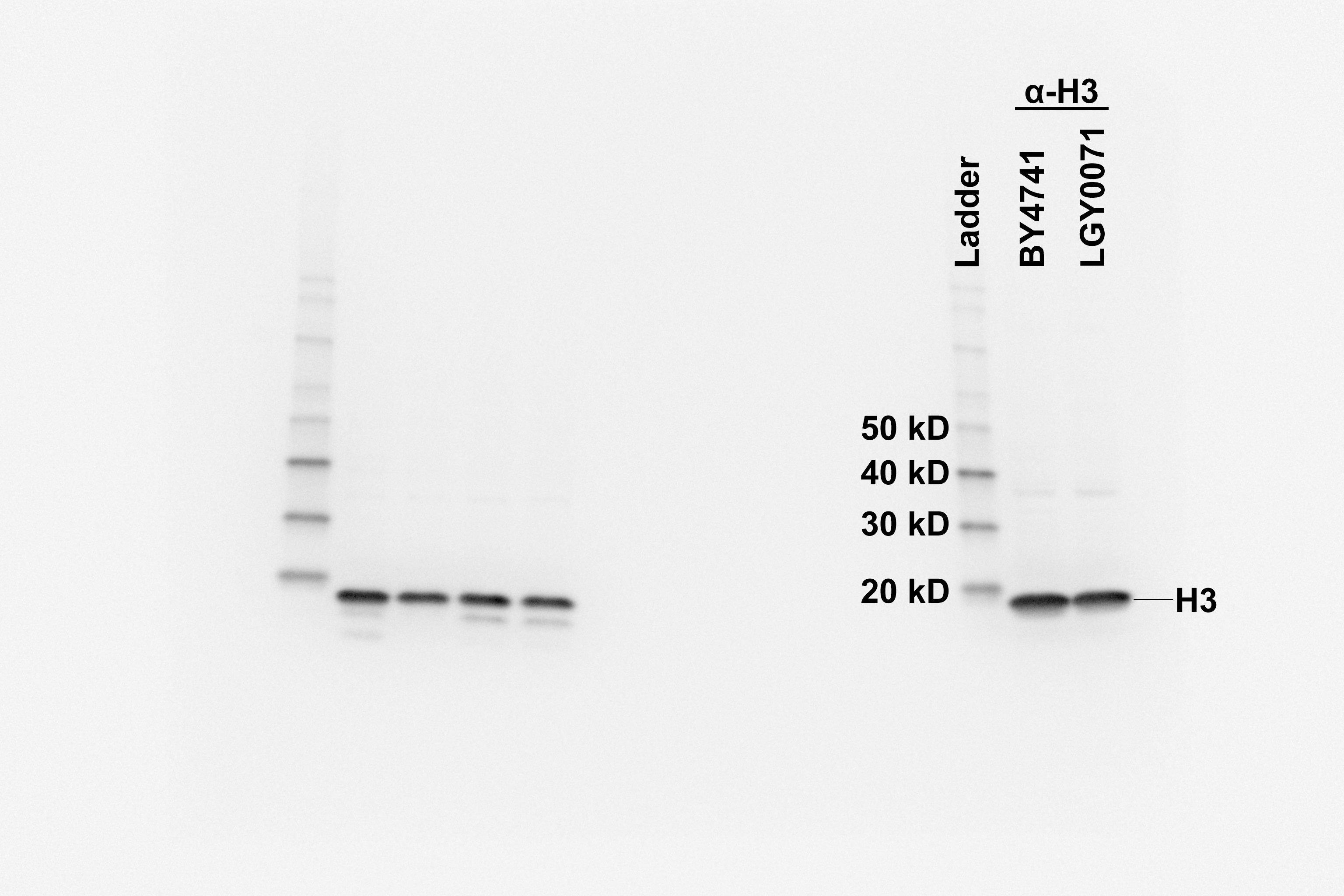

Supplement: Figure 4—figure supplement 8—source data 2. — The unlabeled bands on the left were from an unrelated experiment. [file elife-87672-fig4-figsupp8-data2.zip › Figure 4-Figure Supplement 8-Source Data 2/Figure 4-Figure Supplement 8-Source Data 2-Labelled.png]

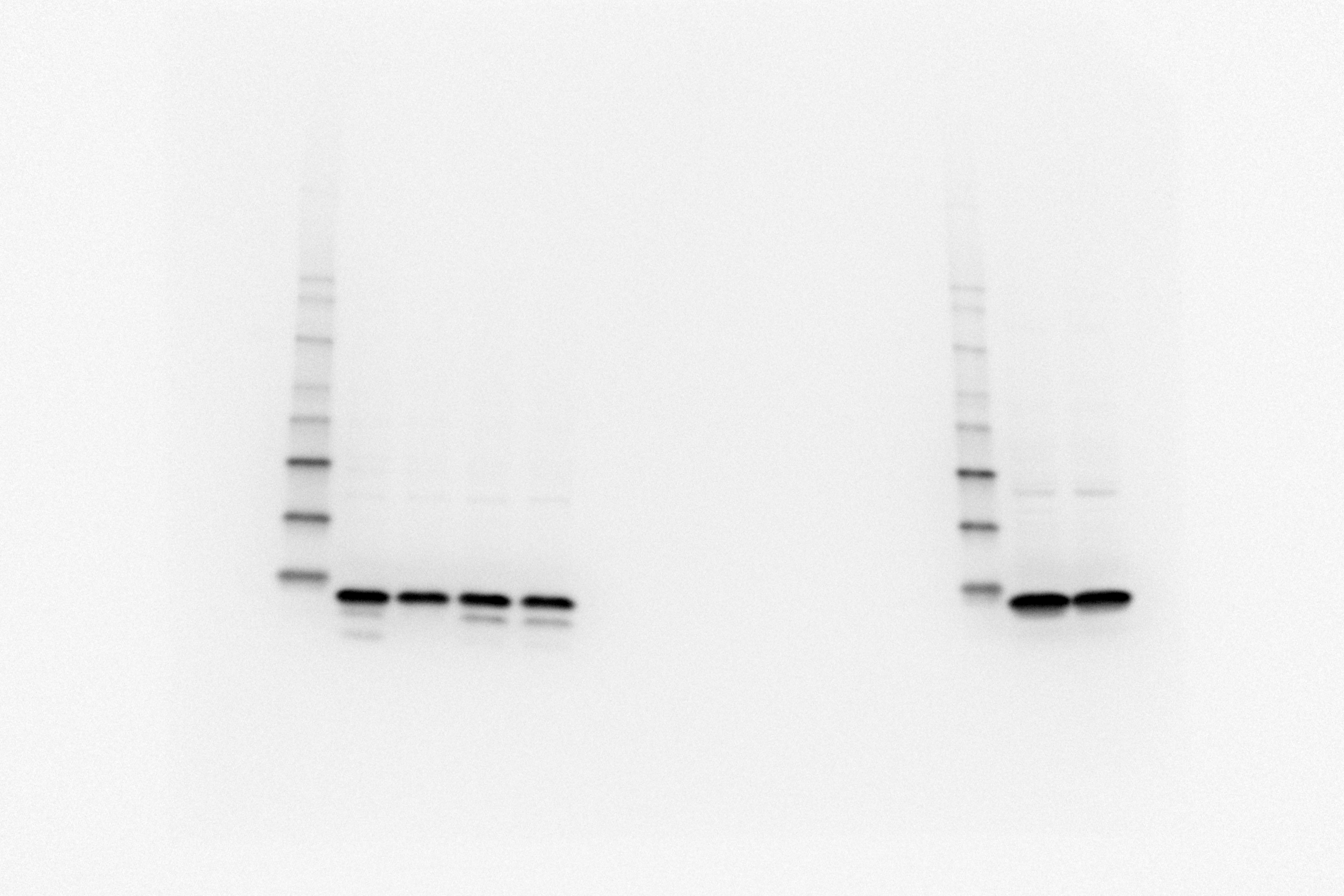

Supplement: Figure 4—figure supplement 8—source data 2. — The unlabeled bands on the left were from an unrelated experiment. [file elife-87672-fig4-figsupp8-data2.zip › Figure 4-Figure Supplement 8-Source Data 2/Figure 4-Figure Supplement 8-Source Data 2-Raw.tif]
